# Smart-glasses-assisted deep learning for in-vehicle CAN intrusion detection

**DOI:** 10.1371/journal.pone.0362367

**Published:** 2026-11-13

**Authors:** Kuo-Yu Tsai, Ting-Jung Huang, Qi-Xiang Wang, Wen-Xin Lee

**Affiliations:** 1 Department of Information Engineering and Computer Science, Feng Chia University, Taichung City, Taiwan; 2 Master’s Program of Information and Communication Security, Feng Chia University, Taichung, Taiwan; Sultan Qaboos University, OMAN

## Abstract

This study presents a smart-glasses-assisted deep learning framework for laboratory proof-of-concept detection and visualization of abnormal in-vehicle Controller Area Network (CAN) traffic. The proposed framework combines CAN traffic classification with a wearable driver-facing display. A mobile application was used to simulate selected benign and malicious CAN traffic, a computer-based detection module performed model inference, and smart glasses displayed the predicted traffic or attack type. The model was trained and evaluated using two publicly available datasets: the Car-Hacking dataset from the Hacking and Countermeasure Research Lab and the CICIoV2024 dataset from the Canadian Institute for Cybersecurity at the University of New Brunswick. The raw CAN data were preprocessed by selecting identifier and payload-related fields, converting hexadecimal values to decimal values, normalizing the inputs, and reshaping consecutive CAN records into image-like samples for convolutional neural network (CNN) classification. Five CNN architectures were compared: Xception, VGG16, VGG19, InceptionV3, and InceptionResNetV2. VGG16 achieved F1-scores of 97.4% on the Car-Hacking dataset and 99.9% on the CICIoV2024 dataset. The results show that CNN-based classification can identify multiple categories of in-vehicle network traffic with high F1-scores in the evaluated datasets. The smart-glasses component demonstrates a proof-of-concept method for displaying detected attack types to drivers or operators. However, the current study did not measure end-to-end latency or conduct a formal driver/user study; therefore, the real-world timing, usability, workload, and distraction effects of the proposed interface require further evaluation.

## Introduction

The Controller Area Network (CAN or CAN bus) was originally designed as a closed communication system for data exchange among electronic components within a vehicle. It enables efficient message transmission between electronic control units (ECUs) and remains a core protocol in modern in-vehicle networks. However, as vehicles become increasingly connected through smart vehicle systems, mobile applications, cloud services, and Internet-enabled devices, the assumptions underlying the original CAN design have changed. In particular, the CAN protocol does not provide built-in encryption or authentication, making it vulnerable to message injection, spoofing, replay, and denial-of-service (DoS) attacks. Early experimental studies demonstrated that compromised in-vehicle components can be used to manipulate safety-relevant vehicle functions and that remote attack surfaces, including wireless and external interfaces, can provide pathways to internal vehicular networks [1,2].

Recent studies and security reports have shown that connected vehicles are exposed to a broad range of cyber threats. In 2022, a group of cybersecurity researchers reported multiple vulnerabilities affecting vehicles from 16 major automakers. These vulnerabilities allowed attackers to remotely unlock vehicles, start or stop engines, modify remote management privileges, and access manufacturer-level functions through associated mobile applications. The reported level of access could also enable attackers to track the real-time locations of approximately 15 million vehicles and issue remote commands [3]. These incidents highlight the increasing importance of cybersecurity in connected vehicles. This need is also reflected in emerging automotive cybersecurity governance frameworks. ISO/SAE 21434 defines engineering requirements for cybersecurity risk management across the lifecycle of road vehicle electrical and electronic systems, while UN Regulation No. 155 requires vehicle manufacturers to address cybersecurity and cybersecurity management systems for vehicle type approval [4,5]. Since attacks on in-vehicle networks may affect safety-critical functions, early detection of abnormal network behavior is important for reducing potential risks to drivers, passengers, and other road users.

Several intrusion detection approaches have been proposed to improve the security of in-vehicle networks. Review studies have categorized CAN intrusion detection methods into frequency-based, statistical, machine learning-based, deep learning-based, and hybrid approaches, and have emphasized that intrusion detection systems (IDSs) are practical defenses because they can often be deployed without redesigning the entire in-vehicle communication architecture [6,7]. More recently, Liu et al. [8] provided a comprehensive survey and evaluation of vehicular IDSs for CAN and ECUs, highlighting that many existing approaches still focus on limited attack categories and face challenges in practical deployment.

Seo et al. [9] proposed a generative adversarial network (GAN)-based intrusion detection model for vehicular networks. Their method converts network data into image representations using one-hot vector encoding and trains the model using the Car-Hacking dataset. The experimental results showed that the first discriminator achieved 100% accuracy, while the second discriminator achieved 98% accuracy. Yang and Shami [10] proposed a CNN-based intrusion detection model using transfer learning and particle swarm optimization (PSO) for hyperparameter tuning. Their model was evaluated using the Car-Hacking and CICIDS2017 datasets and achieved an average accuracy and F1-score of 99.925%. Caivano et al. [11] proposed a multi-class random forest classifier for vehicle intrusion detection and reported 99% accuracy on the Car-Hacking dataset and 99.97% accuracy in experiments involving additional automotive intrusion datasets. Rajapaksha et al. [12] proposed a gated recurrent unit (GRU)-based IDS that uses CAN identifier statistics and threshold values to distinguish normal and abnormal traffic. Their model was evaluated across 16 attack types, including camouflage attacks, and 13 attack types achieved F1-scores above 99%.

Recent studies have further expanded the design space of CAN intrusion detection. Rai et al. [13] evaluated deep learning models, including recurrent neural network variants and VGG16, for CAN bus intrusion detection. Ileri et al. [14] proposed MetaCAN, a machine learning-based IDS optimized using hybrid metaheuristic methods. Sreelekshmi and Aji [15] transformed CAN traffic into graph-based feature images to integrate identifier-based and payload-based information. Cai et al. [16] proposed a LSH-based IDS to reduce computational overhead. Sharmin et al. [17] proposed CAN-BiGRUBERT for predicting vehicle states and attack classes, while Feng et al. [18] introduced a lightweight MambaCNN framework for Internet of Vehicles intrusion detection. These studies indicate that recent CAN IDS research is moving toward multiclass classification, richer data representations, and deployment-aware model design. A summary of representative studies related to vehicle intrusion detection is presented in Table 1.

**Table 1:** Summary of representative related in-vehicle intrusion detection studies.

Study	Method and data source	Main focus	Limitation or gap relevant to this study
Seo et al. [9]	GAN-based IDS; Car-Hacking	Image-based detection using one-hot encoding	Focused on detection; no driver-facing visualization interface
Yang and Shami [10]	Transfer-learning CNN; Car-Hacking and CICIDS2017	CNN model selection and hyperparameter tuning	Does not address driver-facing alert presentation
Caivano et al. [11]	Multi-class random forest; automotive datasets	Multiclass intrusion detection	Focused on classification performance rather than alert delivery
Rajapaksha et al. [12]	GRU and context-aware features; Car-Hacking, ROAD, and Survival Analysis	Normal-data training and identifier statistics	Broader attack coverage but no wearable visualization
Rai et al. [13]	LSTM, GRU, Bi-LSTM, and VGG16; public CAN datasets	Deep-learning comparison for CAN IDS	Demonstrates model potential but does not study HMI presentation
Ileri et al. [14]	Metaheuristic ML; multiple CAN datasets	Optimized multiclass attack detection	Deployment and human alert interaction remain open issues
Sreelekshmi and Aji [15]	Graph feature images; CAN traffic datasets	Identifier-payload representation for IDS	Focuses on model robustness rather than driver notification
Cai et al. [16]	LSH-based IDS; CAN bus traffic	Lightweight IDS design	Does not examine wearable or driver-facing displays
Sharmin et al. [17]	CAN-BiGRUBERT; CAN datasets	State and attack prediction	Highlights need for richer classification targets
Feng et al. [18]	MambaCNN; IoV intrusion datasets	Lightweight deep learning for IoV IDS	Deployment feasibility remains a key evaluation criterion

Deep learning and machine learning have also been applied to image-like or structured data in other engineering domains, including composite material property prediction, landslide susceptibility assessment, geotechnical behavior modeling, and rock constitutive modeling [19–22]. These studies are outside the vehicle-security domain, but they show that CNNs and related learning models are often used when raw signals, spatial data, or structured engineering measurements can be transformed into model-compatible representations. This broader literature supports the use of representation design as an important step when applying learning-based methods to engineering problems.

Although previous studies have achieved high detection performance using machine learning and deep learning methods, most have focused primarily on improving classification accuracy. Less attention has been given to how detection results can be communicated to drivers or operators through an immediate and understandable interface. The problem addressed in this study is therefore not the proposal of a new CNN architecture, but the integration of CAN traffic classification with a wearable visualization interface in a laboratory proof-of-concept system. The objectives of this study are to: (1) preprocess public CAN intrusion datasets into an image-like format suitable for CNN classification; (2) compare multiple CNN architectures and select a model for the proof-of-concept system; (3) implement a simulation workflow in which an Android application sends CAN-like traffic to a computer-based IDS; and (4) display the predicted traffic or attack type on smart glasses. The remainder of the paper is organized as follows. The next section reviews the CAN protocol and summarizes key abbreviations. The proposed system section describes the experimental environment, dataset preprocessing, model training, and wearable visualization interface. The experimental results section reports model performance and simulation outcomes. The discussion section compares the findings with previous work and explains the limitations of the current laboratory setup. The final section concludes the paper and outlines future research directions.

## Review of Controller Area Network

The Controller Area Network (CAN or CAN bus) [23] is a communication protocol developed by Robert Bosch GmbH in the 1980s. It was designed to support efficient and reliable communication among ECUs within vehicles. As the number of ECUs in modern vehicles increased, CAN became an important protocol for reducing wiring complexity while enabling fast data exchange among distributed electronic components. Owing to its robustness, reliability, and real-time communication capability, CAN has been widely adopted not only in the automotive industry but also in other domains, including industrial automation, aviation, and medical equipment.

Data transmission in CAN is performed using structured message frames, commonly referred to as CAN frames or CAN packets. A standard CAN data frame consists of several fields used for synchronization, arbitration, control, data transmission, error detection, acknowledgment, and frame termination. The main components of a standard CAN frame are summarized in Table 2 and described below.

1. **Start of Frame (SOF):** Indicates the beginning of a CAN frame. It consists of a dominant bit with a fixed value of 0, signaling that a node is starting transmission.
2. **Identifier (ID):** Represents the message identifier and determines the arbitration priority of the frame. In CAN arbitration, lower identifier values have higher priority.
3. **Remote Transmission Request (RTR):** Indicates whether the frame is a data frame or a remote frame. A data frame is used to transmit data, whereas a remote frame is used to request data from another node.
4. **Identifier Extension (IDE):** Specifies whether the frame uses the standard 11-bit identifier format or the extended 29-bit identifier format.
5. **Reserved Bit (R0):** A reserved bit in the control field, which is typically fixed to 0 in the standard CAN frame.
6. **Data Length Code (DLC):** Specifies the number of bytes contained in the data field. In classical CAN, the data field length ranges from 0 to 8 bytes.
7. **Data Field:** Contains the payload data transmitted by the sender. Its length is determined by the DLC.
8. **Cyclic Redundancy Check (CRC):** Provides error detection by allowing receiving nodes to verify the integrity of the transmitted frame.
9. **Acknowledgment (ACK):** Indicates whether at least one receiving node has correctly received and validated the frame.
10. **End of Frame (EOF):** Marks the end of the CAN frame.

**Table 2:** Structure of a standard CAN data frame.

Component	Field group	Size	Function
SOF	Start	1 bit	Marks frame start
Arbitration ID	Arbitration	11 bits	Identifies message and priority
RTR	Arbitration	1 bit	Distinguishes data and remote frames
IDE	Control	1 bit	Indicates standard or extended format
R0	Control	1 bit	Reserved bit
DLC	Control	4 bits	Specifies payload length
Data field	Data	0–8 bytes	Contains payload data
CRC	Error check	16 bits	Supports error detection
ACK	Acknowledgment	2 bits	Indicates successful reception
EOF	End	7 bits	Marks frame end

The CAN protocol was originally designed for closed in-vehicle environments, where communication was limited to trusted internal electronic components. However, modern vehicles increasingly interact with external systems, including mobile applications, cloud platforms, roadside infrastructure, global positioning systems, and infotainment services. This expanded connectivity has changed the threat model of in-vehicle networks. Because classical CAN does not provide built-in encryption or authentication mechanisms, malicious nodes or compromised interfaces may inject, replay, or spoof CAN messages. Such attacks can interfere with ECU communication, falsify vehicle status information, and potentially affect safety-critical functions.

These limitations have made CAN security an important research topic. In particular, vehicle IDSs have received increasing attention as a practical defense mechanism for identifying abnormal traffic and detecting potential attacks in in-vehicle networks. By monitoring CAN traffic and detecting deviations from normal communication patterns, IDSs can support early warning and improve the overall cybersecurity of connected vehicles. The abbreviations used throughout this manuscript are summarized in Table 3.

**Table 3:** List of abbreviations.

Abbreviation	Definition
ACK	Acknowledgment
CAN	Controller Area Network
CNN	Convolutional Neural Network
CRC	Cyclic Redundancy Check
DBC	Database for CAN
DLC	Data Length Code
DoS	Denial of Service
ECU	Electronic Control Unit
EOF	End of Frame
GAN	Generative Adversarial Network
GRU	Gated Recurrent Unit
HMI	Human–Machine Interface
HUD	Head-Up Display
ID	Identifier
IDE	Identifier Extension
IDS	Intrusion Detection System
IoV	Internet of Vehicles
PSO	Particle Swarm Optimization
RTR	Remote Transmission Request
SOF	Start of Frame
VGG16	Visual Geometry Group 16-layer network

## Proposed system

This section presents the proposed system, including the experimental environment, input data, model training process, and the functions and interfaces of the mobile application and smart glasses application. The experimental environment describes the hardware and software environment used in the laboratory setup, as well as the overall system architecture. Dataset preprocessing explains the procedures used to convert raw CAN traffic into a suitable input format for the CNN model. Model training introduces the evaluated model architectures and the selected model. The final part presents the mobile application and smart glasses visualization interface.

### Experimental environment

The experimental environment consists of a computer running Windows 11 64-bit, equipped with an Intel i7-13700 processor, an NVIDIA RTX 3090 graphics card, and 64 GB of RAM. The smart glasses used in this study are EPSON Moverio BT-300, while the mobile device is a SAMSUNG Galaxy A50. Android Studio is used as the development platform for both applications. The mobile application is developed using API level 30, whereas the smart glasses application is developed using API level 22.

This experiment simulates an in-vehicle intrusion detection scenario by integrating a deep learning-based detection model with smart glasses in a laboratory proof-of-concept environment. As shown in Fig 1, the system architecture consists of four main components: the attack simulation module, the intrusion detection module, the visual alert module, and the communication environment.

1. **Attack simulation module—Android app as attacker:** An Android smartphone is used to simulate an attacker by running an application that generates synthetic CAN packets. These packets represent selected in-vehicle traffic types, such as benign traffic, DoS attacks, fuzzy attacks, and deception attacks. The generated packets are transmitted to the computer through Wi-Fi, thereby emulating abnormal traffic in a controlled in-vehicle network simulation.
2. **Intrusion detection module—computer as vehicle system:** The computer acts as the vehicle-side processing unit and runs the CNN-based intrusion detection model. The model is trained using the Car-Hacking and CICIoV2024 datasets and is configured to classify the normal and malicious categories available in each dataset. After receiving packets from the Android smartphone, the computer converts the incoming data into the model input format and outputs the corresponding prediction result, including the detected traffic or attack type.
3. **Visual alert module—smart glasses as driver interface:** The prediction result generated by the intrusion detection model is transmitted to the EPSON Moverio BT-300 smart glasses through Wi-Fi. The smart glasses display the detected traffic or attack type and related warning information. The interface was designed to reduce visual clutter and present concise alert information; however, this study did not conduct a formal driver/user study, so its effects on driver attention, workload, field of view, and response behavior require further evaluation.
4. **Communication environment:** All devices in the experiment communicate through a local Wi-Fi network, which is used to simulate the communication path among the attack-generation device, computer-based detection module, and smart glasses display. This setup provides a practical proof-of-concept framework for evaluating intrusion detection and alert visualization, but it is not intended to represent a fully deployed production in-vehicle network.

**Fig 1:**
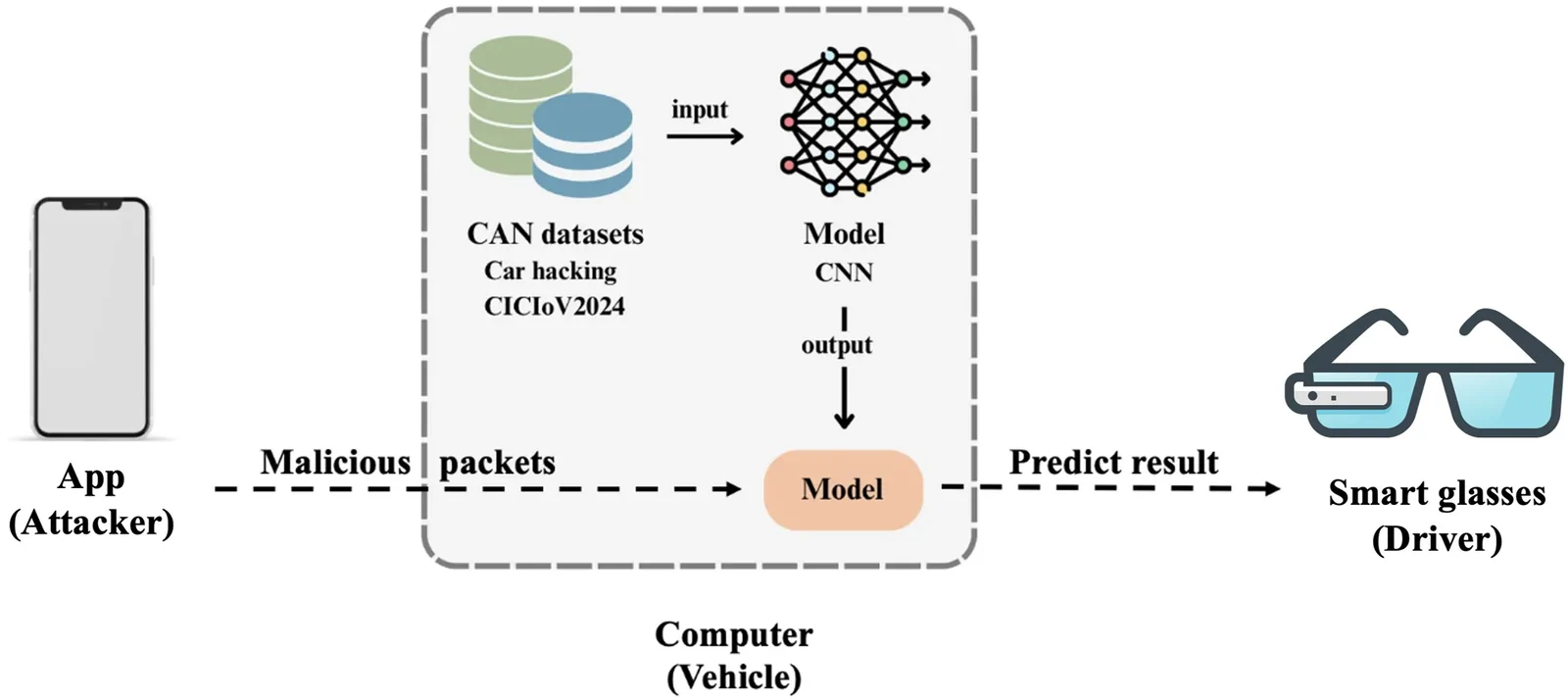
Experimental environment architecture.

### Dataset preprocessing

To support model training, the datasets are processed through a series of preprocessing steps. The preprocessing procedures for the Car-Hacking dataset and the CICIoV2024 dataset are generally similar. Taking the Car-Hacking dataset as an example, the original data format is shown in Fig 2.

**Fig 2:**
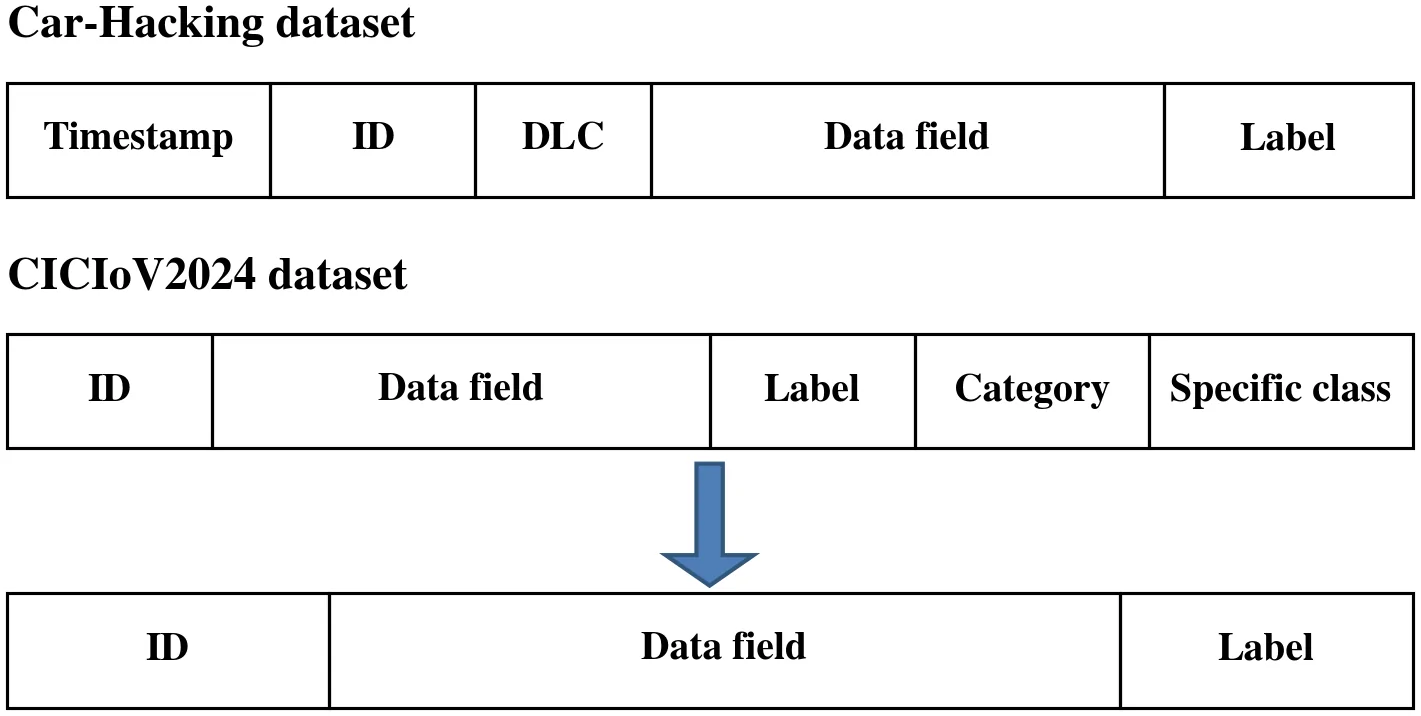
Data format and preprocessing workflow.

First, fields that were not used in the proposed input representation, such as Timestamp and DLC, are removed to reduce dimensionality. The remaining CAN data are then converted from hexadecimal format to decimal format. After conversion, normalization is applied to scale all values to the range between 0 and 1. This step ensures that each feature contributes proportionally during model training and prevents features with larger numerical values from dominating the learning process.

After preprocessing, the data are divided into a training set and a testing set at a ratio of 80:20. To transform CAN traffic into a CNN-compatible input format, every 27 consecutive data entries are combined and reshaped into a single image-like sample. Each generated image is then used as one input sample for model training and testing. The 27-frame window is used as a fixed-length representation for this proof-of-concept study. Because this work does not measure the time required to accumulate 27 frames under a real CAN bus load or the end-to-end processing latency of the full system, the acceptable window size for safety-critical deployment should be evaluated in future work. The overall transformation process from raw CAN data to image-based model input is illustrated in Fig 2.

### Model training

A CNN is employed as the core architecture of the proposed intrusion detection model. Since the early layers of a CNN are generally effective in extracting common visual features, while the deeper layers are responsible for learning task-specific representations and performing classification [24], transfer learning is adopted in this study to improve training efficiency. By using a pre-trained CNN model, the training process can be accelerated while maintaining strong classification performance.

Following the approach proposed by Yang and Shami [10], several well-known CNN architectures are evaluated, including Xception, VGG16, VGG19, InceptionV3, and InceptionResNetV2. These models are compared to identify a suitable architecture for CAN-based intrusion detection under the proposed image-like input representation. Based on the experimental results, VGG16 is selected as the final model because it provides high and stable classification performance across the two evaluated datasets while retaining a relatively regular architecture. VGG16 was originally proposed as a deep convolutional architecture that uses stacked small convolution filters and increased network depth to improve image recognition performance, making it a widely used baseline for transfer learning tasks [25]. The architecture of the selected model is shown in Table 4. The output layer shown in Table 4 corresponds to the Car-Hacking classification setting and can be adapted to the number of classes in the CICIoV2024 dataset.

**Table 4:** Model summary of the selected VGG16-based classifier.

Layer	Output shape	Parameters
InputLayer	(None, 224, 224, 3)	0
Conv2D	(None, 224, 224, 64)	1,792
Conv2D	(None, 224, 224, 64)	36,928
MaxPooling2D	(None, 112, 112, 64)	0
Conv2D	(None, 112, 112, 128)	73,856
Conv2D	(None, 112, 112, 128)	147,584
MaxPooling2D	(None, 56, 56, 128)	0
Conv2D	(None, 56, 56, 256)	295,168
Conv2D	(None, 56, 56, 256)	590,080
Conv2D	(None, 56, 56, 256)	590,080
MaxPooling2D	(None, 28, 28, 256)	0
Conv2D	(None, 28, 28, 512)	1,180,160
Conv2D	(None, 28, 28, 512)	2,359,808
Conv2D	(None, 28, 28, 512)	2,359,808
MaxPooling2D	(None, 14, 14, 512)	0
Conv2D	(None, 14, 14, 512)	2,359,808
Conv2D	(None, 14, 14, 512)	2,359,808
Conv2D	(None, 14, 14, 512)	2,359,808
MaxPooling2D	(None, 7, 7, 512)	0
GlobalAveragePooling2D	(None, 512)	0
Dense	(None, 256)	131,328
Dropout	(None, 256)	0
Dense	(None, 5)	1,285
**Total parameters**		**14,847,301**

## Experimental results and analysis

### Model selection and training results

Five CNN architectures commonly used in related studies were selected for training and evaluation. The experimental results are summarized in Table 5. In this study, the F1-score is used as the primary evaluation metric because it considers both precision and recall, making it suitable for evaluating intrusion detection performance.

**Table 5:** F1-score of the test datasets.

Model	Car-Hacking	CICIoV2024
Xception	75.3%	90.8%
VGG16	97.4%	99.9%
VGG19	96.1%	99.7%
InceptionV3	99.1%	99.8%
InceptionResNetV2	97.5%	99.5%

As shown in Table 5, InceptionV3 achieved the highest F1-score on the Car-Hacking dataset, while VGG16 achieved the best performance on the CICIoV2024 dataset. Although InceptionV3 and InceptionResNetV2 demonstrated strong classification performance, VGG16 was selected for the implemented proof-of-concept because it provided consistently high performance across both datasets and is a widely used transfer learning baseline. This selection should not be interpreted as evidence that VGG16 is optimal for resource-constrained in-vehicle deployment, because this study did not benchmark inference time, memory use, or end-to-end communication latency.

Therefore, VGG16 was used as the final model in this study. It achieved an F1-score of 97.4% on the Car-Hacking dataset and 99.9% on the CICIoV2024 dataset, demonstrating high classification performance in the tested data settings. Because this study also evaluates the proposed model on CICIoV2024, recent empirical studies on this dataset are relevant for interpreting the results. Palma et al. [26] evaluated machine learning models on imbalanced CICIoV2024 data, while Le and Alsmadi [27] emphasized that preprocessing, data duplication, and evaluation settings can affect reported performance. Therefore, the high F1-scores reported here should be interpreted together with the preprocessing workflow and data-splitting assumptions. To place the reported results in the context of previously published work, a comparison of representative in-vehicle intrusion detection studies is provided in Table 6.

**Table 6:** Comparison with previously published in-vehicle intrusion detection studies.

Study	Dataset and method	Reported performance	Comparison note
Seo et al. [9]	Car-Hacking; GAN-based IDS	100% and 98% discriminator accuracy	High detection performance; no smart-glasses visualization
Yang and Shami [10]	Car-Hacking and CICIDS2017; optimized CNN	99.925% average accuracy and F1-score	Strong CNN baseline closely related to this study
Caivano et al. [11]	Car-Hacking and other automotive datasets; random forest	99% and 99.97% accuracy in reported experiments	Machine-learning baseline with multiclass classification
Rajapaksha et al. [12]	Car-Hacking, ROAD, and Survival Analysis; GRU-based IDS	F1-score above 99% for 13 of 16 attack types	Includes advanced attack types and latency discussion
Rai et al. [13]	Public CAN datasets; LSTM, GRU, Bi-LSTM, and VGG16	Strong attack-wise performance; VGG16 F1-score lower for fuzzy attacks	Supports use of VGG16 but also shows attack-wise variability
Ileri et al. [14]	Multiple CAN datasets; MetaCAN	Improved multiclass attack detection through optimization	Emphasizes optimized feature engineering and model tuning
Sreelekshmi and Aji [15]	CAN traffic datasets; graph-based feature images	Evaluated for accuracy and latency	Related to the image-based representation used here
Sharmin et al. [17]	CAN datasets; CAN-BiGRUBERT	Higher accuracy and F1-score than baseline models	Extends IDS toward joint vehicle-state and attack-class prediction
Feng et al. [18]	IoV intrusion datasets; MambaCNN	Lightweight IDS design	Highlights the importance of deployment feasibility
This study	Car-Hacking and CICIoV2024; VGG16 with smart-glasses display	F1-score: 97.4% and 99.9%	Adds a proof-of-concept wearable visualization interface; no latency or user study was conducted

The confusion matrices of the test results are shown in Figs. 3 and 4. According to the confusion matrix of the Car-Hacking dataset, the model shows a higher tendency to misclassify DoS and fuzzy attacks. This may be because both attack types generate a large number of abnormal CAN packets within a short period of time, resulting in similar traffic patterns. As a result, the model may have difficulty distinguishing their feature representations. In contrast, the confusion matrix of the CICIoV2024 dataset shows more stable classification results, indicating that the selected model can effectively identify different traffic categories in this dataset under the adopted preprocessing and data split.

**Fig 3:**
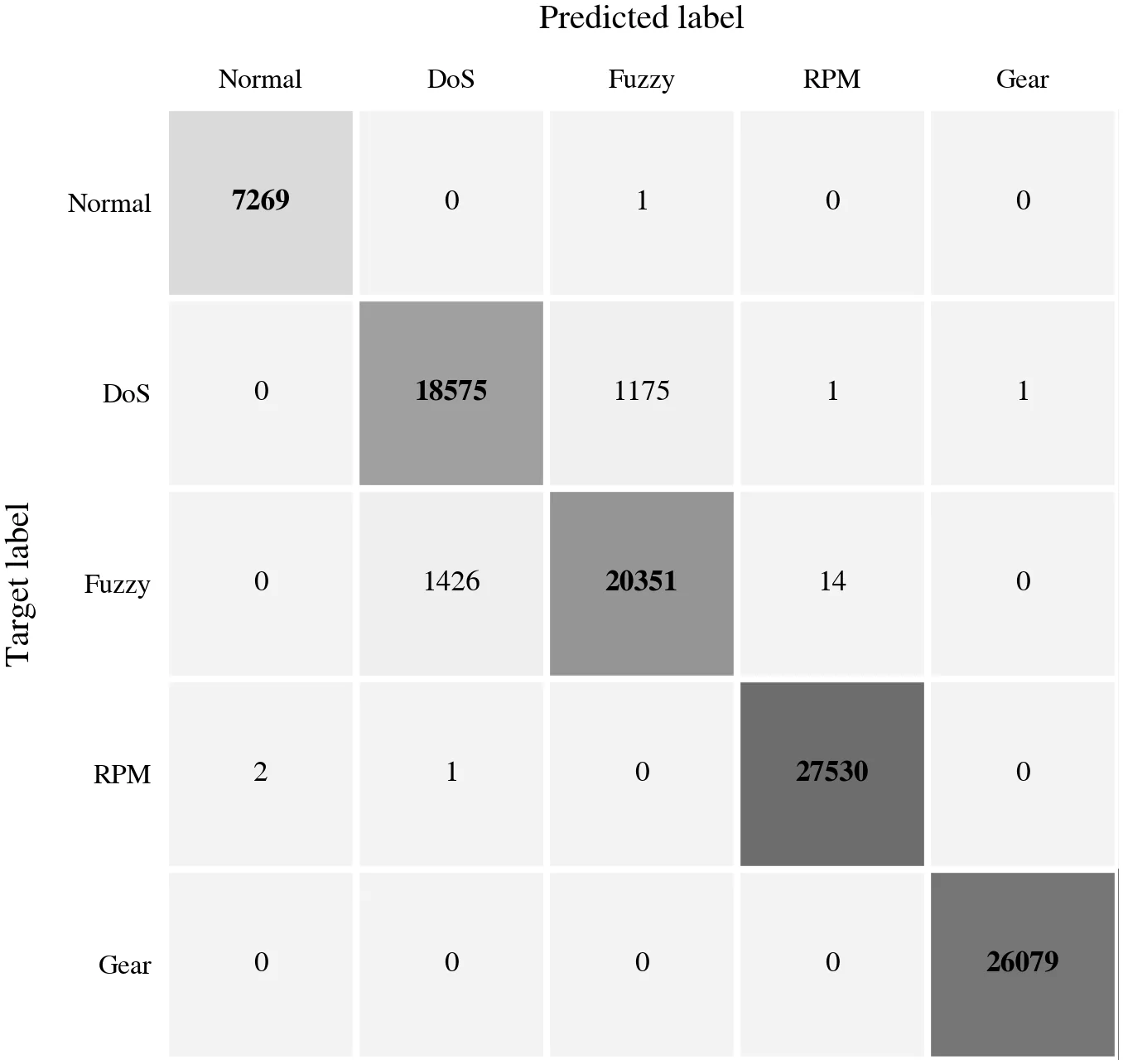
Confusion matrix of the Car-Hacking test dataset.

**Fig 4:**
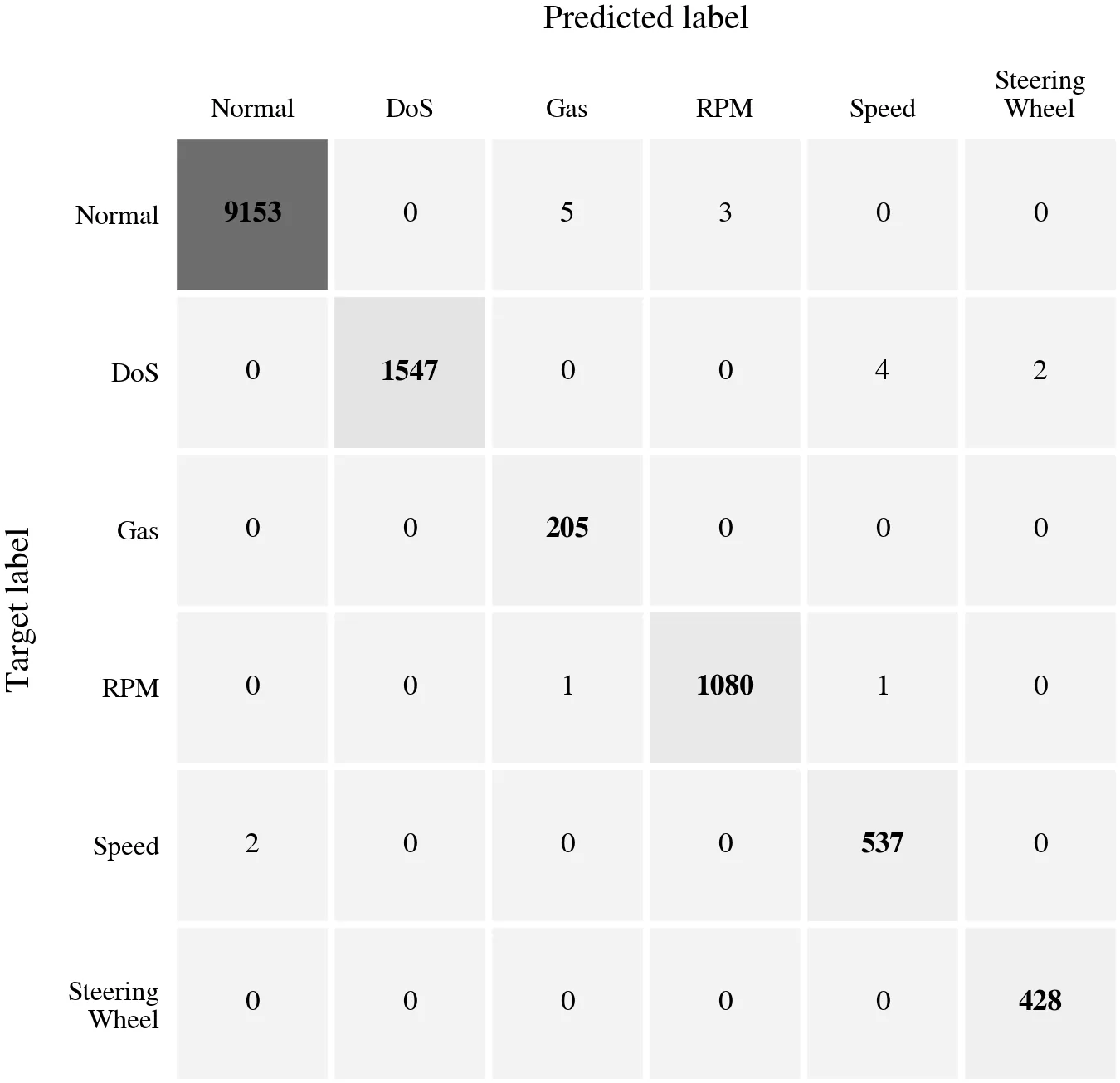
Confusion matrix of the CICIoV2024 test dataset.

In the system simulation, a mobile phone is used to emulate an attacker, as shown in Fig 5. The mobile application allows the user to select a dataset, such as the Car-Hacking dataset, and specify the type of traffic to be generated, including benign traffic, DoS attacks, and other attack types. After selecting the desired traffic type, the user can press the “Attack” button to start the simulation. The mobile application then sends the corresponding CAN packets to the computer through a Wi-Fi network.

**Fig 5:**
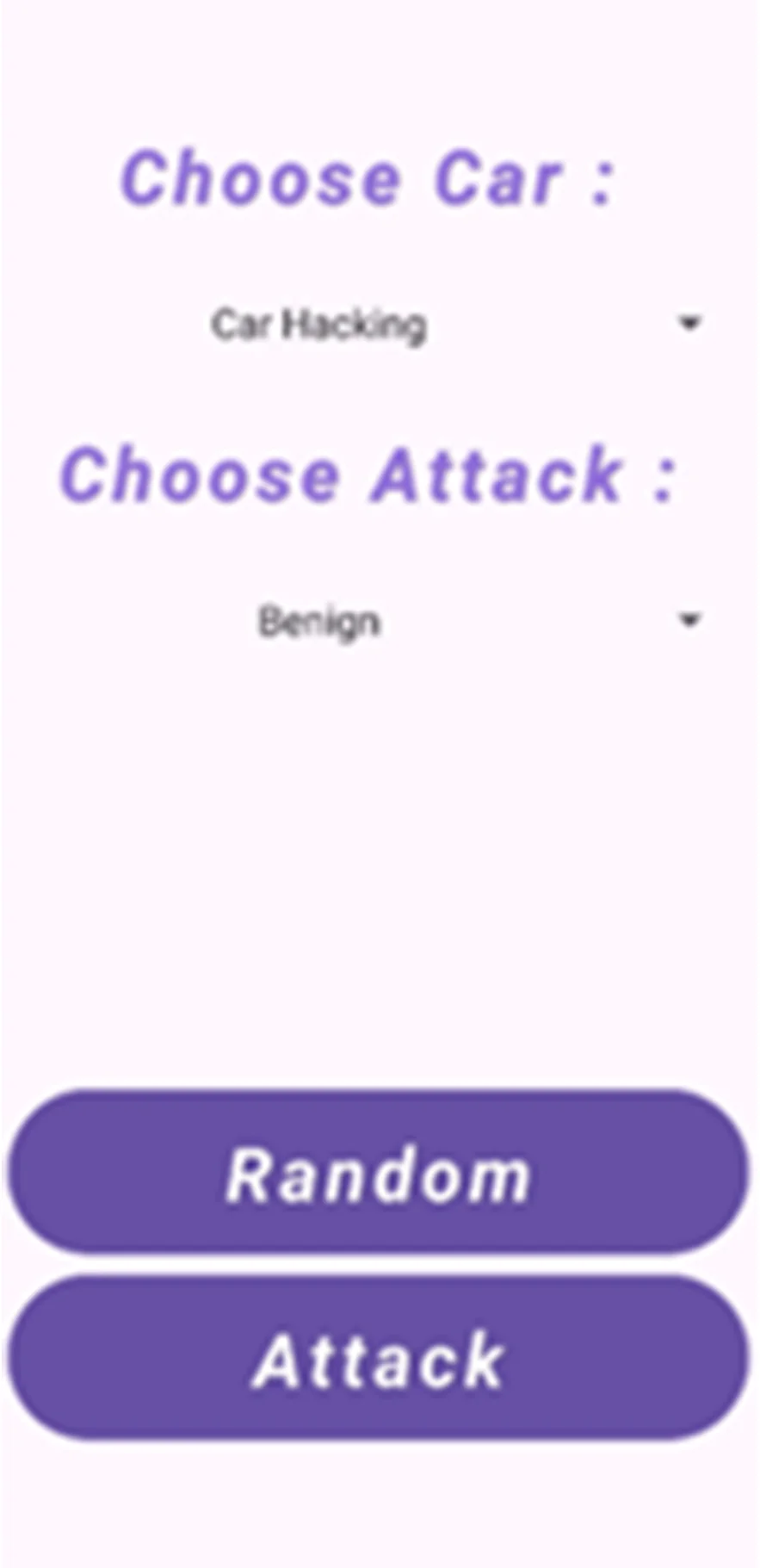
Simulating an attacker’s mobile app.

The computer serves as the in-vehicle intrusion detection module. After receiving the incoming packets, it processes the data and performs classification using the trained CNN model. The prediction result is then transmitted wirelessly to the smart glasses, allowing the detected traffic status or attack type to be displayed in the proof-of-concept interface.

Because driver-facing displays can both support and distract the driver, the alert interface was designed to provide concise warnings with limited visual clutter. Prior work on head-up displays has shown that HUDs may reduce the time required to view driving-related information compared with head-down displays, but they may also attract visual attention because they appear in the driver’s field of view [28]. Recent HMI studies also suggest that alert modality, timing, sensitivity, and display location should be considered together when designing driver-facing warnings [29]. Therefore, the proposed smart glasses interface uses a simple visual alert rather than a complex or information-dense notification. However, this design choice was not validated through a formal driver/user study in the present work.

The visualization interface of the smart glasses changes according to the prediction result:

1. **Normal traffic:** As shown in Fig 6, when the incoming traffic is classified as benign, the smart glasses display only basic driving-related information, such as the current temperature and time. No additional warning message is presented. This design is intended to keep the display simple during normal traffic conditions, but its effect on driver attention requires empirical evaluation.
2. **Malicious traffic:** As shown in Fig 7, when abnormal traffic such as a DoS attack is detected, the smart glasses display a warning message indicating the detected attack type at the top of the screen, such as “-DoS-”. In addition, a warning border appears around the display and flashes by changing between bright and dark states. This visual effect is intended to attract attention while keeping essential information, such as temperature and time, visible; nevertheless, the distraction potential and workload effects require formal testing.

**Fig 6:**
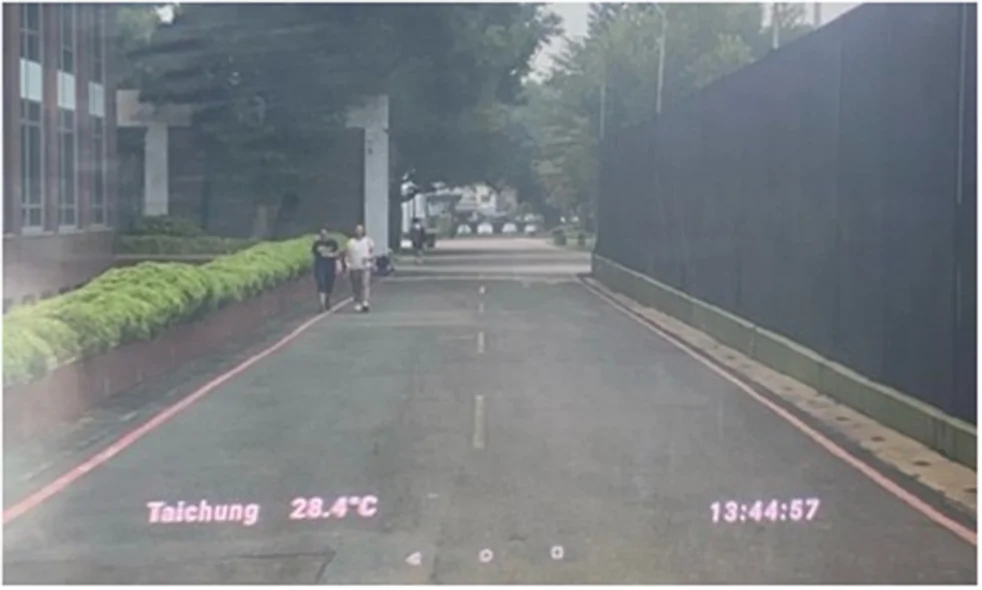
Normal driving vision. This figure was created by the authors during the smart-glasses simulation experiment.

**Fig 7:**
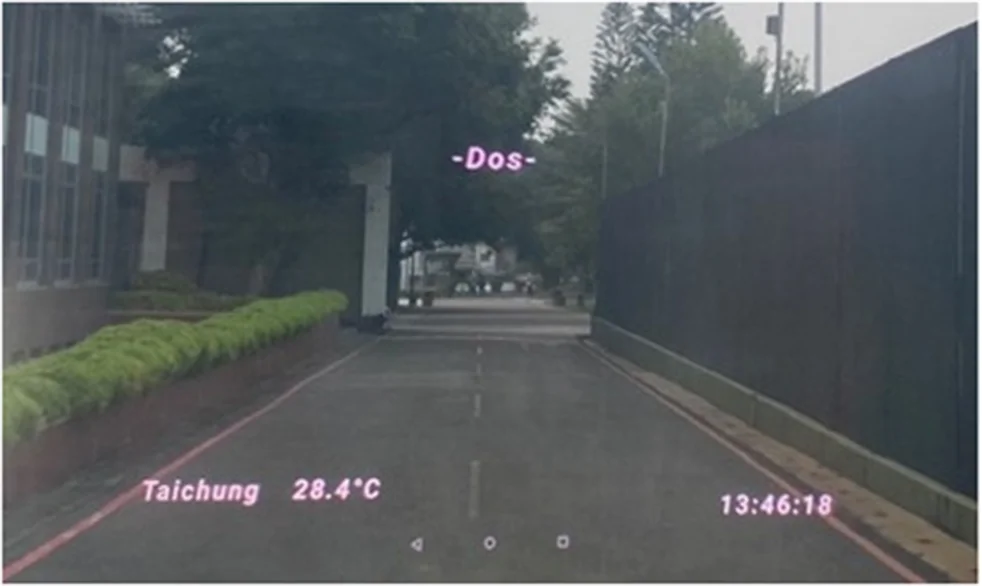
Driving vision in the attack state. This figure was created by the authors during the smart-glasses simulation experiment.

This proof-of-concept visualization mechanism shows how intrusion alerts can be delivered to a wearable display. The experimental results demonstrate the feasibility of integrating a deep learning-based vehicle intrusion detection model with a smart glasses interface in a controlled laboratory setting. However, the current implementation does not establish real-world deployment readiness, end-to-end response timing, or driver safety benefits.

## Discussion

The results show that the proposed CNN-based classifier can achieve high F1-scores on two public CAN intrusion datasets. The VGG16 model achieved 97.4% on the Car-Hacking dataset and 99.9% on the CICIoV2024 dataset, while InceptionV3 achieved the highest F1-score on the Car-Hacking dataset. These findings indicate that image-like representations of CAN traffic can be effective for dataset-based classification. However, the difference between the best-performing model on Car-Hacking and the model selected for the proof-of-concept system also shows that model selection depends on the target dataset, the implementation objective, and the intended deployment constraints.

Compared with previous studies, the contribution of this study is primarily system-level integration rather than a new deep learning architecture. Prior work has proposed GAN-based, CNN-based, random forest-based, GRU-based, graph-based, and lightweight models for CAN intrusion detection [9–12,15,16,18]. The present study extends this line of work by linking CNN-based detection outputs to a smart-glasses visualization interface. This integration is intended to explore how detection results could be communicated to drivers or operators in a compact visual form. Therefore, the novelty lies in the laboratory proof-of-concept workflow that combines attack simulation, CAN traffic classification, wireless result transmission, and wearable visualization.

The results should be interpreted with caution for several reasons. First, the datasets used in this study are public benchmark datasets, and the reported F1-scores depend on the adopted preprocessing, data representation, and train-test split. Recent studies on CICIoV2024 have shown that class imbalance, duplicated records, and preprocessing settings may influence measured performance [26,27]. Future evaluations should therefore include attack-wise metrics, cross-dataset validation, reduced-duplication settings, and tests under traffic distributions that more closely reflect real vehicle operation.

Second, the current setup uses a smartphone, a desktop computer, and smart glasses communicating through Wi-Fi. This configuration is useful for a laboratory proof of concept, but it does not fully reproduce the strict timing constraints of a production in-vehicle CAN bus. The current study did not collect system-level timing measurements, including the time required to accumulate the 27-frame input window, preprocessing time, model inference time, communication delay, display-rendering delay, or total end-to-end response time. A concrete evaluation protocol for future deployment should measure these components separately and jointly. Specifically, future experiments should record: (i) the 27-frame accumulation time from the first CAN frame to the 27th frame; (ii) preprocessing and normalization time; (iii) CNN inference time on the target hardware; (iv) input communication delay from the traffic source to the detection module; (v) output communication delay from the detection module to the display device; and (vi) display-rendering delay on the driver-facing interface. The total alert latency should then be reported as the sum of these components, together with the mean, median, 95th-percentile latency, jitter, packet loss rate, and hardware resource consumption.

A practical deployment also requires balancing latency, computational requirements, and detection accuracy. A shorter input window or a smaller model may reduce delay and memory consumption, but it may also remove temporal context that helps distinguish similar attacks such as DoS and fuzzy traffic. Conversely, a larger input window or heavier model may improve classification robustness but increase the time required to generate an alert. The selected VGG16-based model contains approximately 14.8 million parameters and uses 224×224 image inputs. Therefore, compression, pruning, quantization, model distillation, or alternative lightweight architectures should be considered before deployment in resource-constrained vehicle hardware.

Third, communication reliability is another deployment limitation. Wi-Fi was used in this work because it is convenient for laboratory prototyping and provides sufficient bandwidth for exchanging simulated packets and alert messages. However, Wi-Fi can be affected by interference, congestion, and coverage loss. Bluetooth may reduce energy consumption and support short-range wearable communication, but it may introduce lower bandwidth and different latency characteristics. Cellular communication could support remote monitoring, but it may add network-dependent delay and service-coverage constraints. Direct local processing on a vehicle-side edge device would remove part of the wireless communication delay, but it would increase the computational and integration requirements of the in-vehicle hardware. During connectivity loss, a future system should avoid treating missing alerts as normal operation. Instead, it should log the event locally, buffer pending alerts when possible, and display a communication-unavailable or degraded-mode warning until the detection and alert channels are restored.

Fourth, the smart-glasses interface was not evaluated using a formal HMI or driver study. Although the interface was designed to be simple, the study did not measure driver reaction time, visual attention, distraction, acceptance, or workload. HMI research indicates that alert modality, timing, sensitivity, and display location can influence driver attention and performance [28,29]. Future studies should therefore conduct controlled user experiments, simulator-based driving studies, or on-road evaluations before making claims about non-distraction, improved driver response, or safety benefits.

Finally, the current attack coverage is limited. The experiments focus mainly on attack categories represented in the Car-Hacking and CICIoV2024 datasets, such as DoS, fuzzy, spoofing, and deception-related attacks. Other attack types, including replay, masquerade, and camouflage attacks, should be included in future experiments. State-aware classification, as explored in recent CAN IDS studies [17], may also help distinguish the operational context in which an abnormal packet appears.

## Conclusions and future work

In this study, we implemented a CNN-based vehicle intrusion detection model and integrated it with smart glasses as a proof-of-concept medium for displaying detection results. The detected traffic or attack type is visually presented on the screen of the smart glasses, allowing the system to demonstrate a driver-facing alert workflow in a laboratory setting. The model was trained and evaluated using two public datasets, Car-Hacking and CICIoV2024. The selected VGG16 model achieved F1-scores of 97.4% and 99.9% on the Car-Hacking and CICIoV2024 datasets, respectively.

The experimental results also indicate several limitations and directions for future work. In terms of model training, distinguishing between DoS and fuzzy attacks remains challenging because these attacks share similar traffic characteristics. This suggests that further improvements are needed to enhance the model’s ability to discriminate between attack types with overlapping features. Moreover, this study focuses mainly on attack types represented in the selected datasets. Other common in-vehicle intrusion methods, such as replay attacks, masquerade attacks, and more sophisticated camouflage attacks, were not fully examined in the current experiments and should be further investigated.

From the perspective of deployment feasibility, the current system should be regarded as a laboratory proof of concept rather than a validated production-ready in-vehicle IDS. Future work should measure inference time, preprocessing time, communication latency, display-rendering delay, and total end-to-end response time using the evaluation protocol described above. It should also investigate whether the 27-frame input window can be collected and processed within an acceptable detection window under real CAN bus traffic conditions. In addition, lightweight model design, model compression, and deployment on vehicle-grade edge hardware should be evaluated. Future prototypes should further compare Wi-Fi, Bluetooth, cellular communication, and direct local processing to determine how different communication modes affect latency, reliability, energy consumption, and system behavior during connectivity loss.

From the perspective of driver-facing visualization, smart glasses may provide a potential alternative for presenting cybersecurity alerts, but important issues such as wearing comfort, user acceptance, visual distraction, field-of-view effects, and compliance with traffic regulations in different countries require further evaluation. Future interface designs should examine whether visual-only alerts are sufficient or whether multimodal alerts, such as combining visual cues with auditory or haptic feedback, can improve driver response without increasing workload [28,29]. Alternative display platforms, such as windshield-based HUDs or vehicle infotainment systems, may also be considered. These improvements may help future systems provide more actionable and empirically validated support during abnormal in-vehicle network events.
